# *Nauclea orientalis* (L.) Bark Extract Protects Rat Cardiomyocytes from Doxorubicin-Induced Oxidative Stress, Inflammation, Apoptosis, and DNA Fragmentation

**DOI:** 10.1155/2022/1714841

**Published:** 2022-02-14

**Authors:** Jayasinghe A. N. Sandamali, Ruwani P. Hewawasam, Kamani A. P. W. Jayatilaka, Lakmini K. B. Mudduwa

**Affiliations:** ^1^Department of Medical Laboratory Science, Faculty of Allied Health Sciences, University of Ruhuna, 80000, Sri Lanka; ^2^Department of Biochemistry, Faculty of Medicine, University of Ruhuna, 80000, Sri Lanka; ^3^Department of Pathology, Faculty of Medicine, University of Ruhuna, 80000, Sri Lanka

## Abstract

The therapeutic efficacy of anthracycline antibiotic, doxorubicin (Dox), is hampered due to the dose-dependent cardiotoxicity. The objective of the study was to explore the counteraction of aqueous bark extract of *Nauclea orientalis* in Dox-induced cardiotoxicity in Wistar rats. The acute and subchronic toxicity study performed with 2.0 g/kg of the plant extract revealed biochemical and haematological parameters to be within the physiological range, and no histological alterations were observed in any organs isolated. Screening of plant extract for the protection of the myocardium from Dox-induced oxidative stress, inflammation, and apoptosis was performed on five groups of rats: control, plant extract control, Dox control (distilled water (D.H_2_O) 2 weeks + on the 11^th^ day single injection of Dox, 18 mg/kg), plant + Dox (2.0 g/kg plant extract 2 weeks + on the 11^th^ day Dox, 18 mg/kg), and positive control, dexrazoxane. A significant increase in cardiac biomarkers and lipid peroxidation (*p* < 0.001) and a significant decrease in antioxidant parameters (*p* < 0.001) were observed in the Dox control group. All these parameters were reversed significantly (*p* < 0.05) in the plant-pretreated group. The histopathological assessment of myocardial damage provided supportive evidence for the biochemical results obtained. Inflammatory markers, myeloperoxidase, expression of TNF*α* and caspase-3, and DNA fragmentation (TUNEL positive nuclei) were significantly elevated (*p* < 0.05), and expression of Bcl-2 was significantly decreased (*p* < 0.05) in the Dox control; however, all these parameters were significantly reversed in the plant extract-treated group. In conclusion, the aqueous bark extract of *Nauclea orientalis* (2.0 g/kg) has the ability to attenuate the Dox-induced oxidative stress, inflammation, apoptosis, and DNA fragmentation in Wistar rats.

## 1. Introduction

Doxorubicin (Dox), the anthracycline antibiotic, is a widely accepted chemotherapeutic agent in the treatment of diverse malignancies [1]. Unfortunately, its long-term usage is restricted by the multiorgan toxicity including severe cardiotoxicity [2]. Dox-induced dose-dependent cardiotoxicity could be manifested by various changes within the heart like cardiac arrhythmias, electrocardiographic alterations, permanent degenerative cardiomyopathy, and congestive heart failure [3]. Several molecular mechanisms have been identified in the pathogenesis of acute and chronic Dox-induced cardiotoxicity including oxidative stress, altered iron metabolism, dysregulation in calcium homeostasis, structural alterations in sarcomeres, modulation of gene expression, and apoptosis [4]. However, one of the foremost important mechanisms is the oxidative stress caused by the generation of free radicals which subsequently cause the lipid peroxidation, reduction of sulfhydryl groups, and depletion of antioxidant enzymes [3]. Additionally, inflammation, apoptosis, and impairment of DNA arise in the myocardium.

The main reason for oxidative stress caused by Dox is the imbalance between reactive oxygen species and the endogenous antioxidant defence system [4]. Radical formation in Dox-induced cardiotoxicity occurs by two main pathways: a nonenzymatic pathway via iron and an enzymatic pathway via mitochondrial respiratory chain [5]. Dox is converted to a Dox semiquinone form by reduced flavoenzymes such as nicotinamide adenine dinucleotide phosphate hydrogen- (NADPH-) cytochrome P450 reductase [5, 6]. This reduced form is in a position to make a complex with iron (Fe^2+^) which has the ability to impulsively reduce molecular oxygen to superoxide. In the enzymatic pathway, free oxygen radicals are also produced when Dox gets reduced at complex I of the electron transport chain by accepting electrons from nicotinamide adenine dinucleotide (NADH) or NADPH [5]. The redox cycling that happens might also be very harmful since a small amount of Dox was found to be adequate for the formation of the large amount of superoxide radicals. As Dox has high affinity to cardiolipin, Dox enters the mitochondria and suppresses respiratory chain by binding to cardiolipin, which is identified as a cardiac-specific, polyunsaturated fatty acid-rich phospholipid found within the mitochondrial internal membrane [5, 7]. Dox is also capable of reducing the action of cardiac enzymes like glutathione S-transferase, superoxide dismutase (SOD), and catalase. Moreover, the myocardial tissues are more vulnerable to be injured by Dox-induced free radicals because the antioxidants available in the heart tissues are low in comparison to other organs [7].

Furthermore, generation of ROS results in activation of the mitochondria-mediated apoptotic signalling pathway which consequently activates caspase-3-mediated intrinsic cardiomyocyte apoptosis [8]. In addition to the activation of extrinsic apoptotic pathway, death ligands like tumour necrosis factor *α* (TNF*α*) also contribute to the apoptosis in cardiomyocytes.

As Dox remains a highly effective anticancer drug for various malignancies, several therapeutic approaches are tested to scale back the occurrence of cardiotoxicity including the administration of defensive agents like antioxidants, iron chelators, and radical scavengers [4]. *Nauclea orientalis* (L.) L. that belongs to the family Rubiaceae is a useful medicinal plant which has a high antioxidant effect. Consistent with previous reports, the root and the bark of this plant contain constituents such as *β*-sitosterol and phenolic substances which play a crucial role in scavenging free radicals in addition to the metal chelating and anticarcinogenic properties [9]. A study led by Daoa et al. has also revealed that the bark of *Nauclea orientalis* (L.) L. has significant DPPH radical scavenging and inhibitory action on lipid peroxidation [10]. Therefore, the aqueous bark extract of *Nauclea orientalis* (L.) L. was selected to determine the cardioprotective effect against Dox-induced cardiotoxicity in Wistar rats.

## 2. Methods

### 2.1. Collection of *Nauclea orientalis* (L.) L. Bark

The identified bark of *Nauclea orientalis* (L.) L. consistent with the study of Jayaweera [11] was collected from the Southern province, Sri Lanka, and authenticated at the National Herbarium, Royal Botanical Gardens, Peradeniya, Sri Lanka.

### 2.2. Pharmacognostic Standardization

#### 2.2.1. Determination of Physicochemical Parameters and Phytochemical Profile

Heavy metal analysis, extractable matter, and moisture content were determined following WHO guidelines [12]. Morphological features were examined using a microscope following WHO (2011) guidelines on quality control and standardization of plant materials [13].

D.H_2_O or organic extraction of the *Nauclea orientalis* (L.) L. bark was used for the phytochemical screening tests in the identification of compounds including glycosides (anthracene, cyanogenic, and cardenoloid), polyphenols, flavonoids, alkaloids, saponin, tannins, reducing sugars, and proteins [14, 15].

### 2.3. Determination of Total Polyphenol Content and In Vitro Antioxidant Activity of *Nauclea orientalis* (L.) L. Bark

#### 2.3.1. Preparation of Aqueous Extract

A *Nauclea orientalis* bark (2.50 g) was dried at 40°C and coarsely ground. Then it was added to D.H_2_O (60 mL) and extracted by refluxing for 3 h. Then the mixture was kept in a rotary evaporator for 1 h at 100 rpm. After that, the mixture was filtered through muslin cloth, and the filtrate was concentrated through evaporation on a water bath at 100°C. The final extract was weighed (0.33 g, percentage yield was 13.2%) and stored. Subsequent assays were performed on a concentration series (1-500 *μ*g/mL) of the extract.

#### 2.3.2. Estimation of Total Polyphenol Content

The aqueous bark extract of *Nauclea* was subjected to determine the polyphenol content based on the spectrophotometric method using the Folin-Ciocalteu reagent [16]. The plant extract (1.0 mL) was added into a solution mixture containing 95% ethanol, D.H_2_O, and the newly prepared 50% Folin-Ciocalteu reagent. After five minutes, the mixture was incubated with sodium carbonate (Na_2_CO_3_) at 27°C for one hour. The absorbance was determined (725 nm), and the results were mentioned as milligrams of gallic acid equivalent per gram of extract dry weight (mgGAE/g dw).

#### 2.3.3. 2,2-Diphenyl-1-Picrylhydrazyl (DPPH) Free Radical Scavenging Activity

The method of Bhuiyan et al. [17] was modified to assess the DPPH radical scavenging activity of the aqueous bark extract of *Nauclea*. The concentration series of the bark extract and the standard (L-ascorbic acid) were incubated in 0.004% DPPH solution (3.0 mL) at 25°C, and the absorbance was measured (517 nm). The absorbance of the control was obtained from 0.004% DPPH solution. The radical scavenging ability (percentage inhibition of free radicals) was calculated by the below-mentioned equation.

(1) $$\begin{array}{c} \text{Radical scavenging activity}\left(\%\right)=\left[\left(\text{Abs control}–\text{Abs test}\right)\right]/\left.\left(\text{Abs control}\right)\right]\times 100. \end{array}$$

The IC_50_ value (concentration of the plant extract or standard required to inhibit DPPH radical formation by 50%) was finally calculated to measure the antioxidant activity of the aqueous bark extract of *Nauclea orientalis*.

#### 2.3.4. Nitric Oxide (NO) Radical Scavenging Activity

Sodium nitroprusside which has the ability to react with oxygen generates nitric oxide; hence, it is used as the NO^•^ generator. The NO radical scavenging activity of *Nauclea* bark was assessed by the modified Griess reaction [18]. The buffered sodium nitroprusside (10 mM) was incubated with the concentration series of aqueous plant extract (1 mL) and the L-ascorbic acid (standard) at 25°C for 180 min. Then they were mixed with the Griess reagent in equal volume. The absorbance of the pink colour formed during the reaction was measured by a spectrophotometer (550 nm). Radical scavenging activity was estimated by Equation (1).

#### 2.3.5. Ferric Reducing Antioxidant Power (FRAP) Assay

The FRAP assay was performed based on the method published by Zahin et al. [19]. In the reaction, Fe^3+^(CN^−^)_6_ is reduced to Fe^2+^(CN^−^)_6_, and the addition of excess ferric ions in the medium develops a Perl's Prussian blue complex. The concentration series of the aqueous bark extract was added into the mixture of potassium ferricyanide (1%, K_3_Fe(CN_6_)) and the phosphate buffer (pH 6.6) and incubated for 20 min (at 50°C). After the addition of 10% trichloroacetic acid and 0.1% FeCl_3_, the mixture was again incubated at room temperature for 20 min, and absorbance was measured (700 nm).

### 2.4. Experimental Animals

Wistar albino rats (6-8 weeks old), weighing 175 ± 25 g, were procured from the Medical Research Institute (MRI), Sri Lanka. The rats were housed in stainless-steel cages in a controlled environment, temperature at 23 ± 2°C, and humidity 55 ± 5% under a 12-hour light-dark cycle. They were fed with a standard laboratory diet of rat pellets and water ad libitum. The animal experiments were followed obeying the CIOMS international guiding principles [20] under the approval of the Ethics Committee of the Faculty of Medicine, University of Ruhuna, Sri Lanka.

### 2.5. Dose-Response Effect of Aqueous Bark Extract of *Nauclea orientalis* (L.) L. against Dox-Induced Cardiotoxicity In Vivo

The following treatment protocol was followed in seven groups of 10 animals in each: group I (control): oral dose of D.H_2_O (10 mL/kg) for 2 weeks and single intraperitoneal (IP) injection of saline on the 11^th^ day; group II (Dox control): oral dose of D.H_2_O (10 mL/kg) for 2 weeks and single IP injection of Dox (18 mg/kg) on the 11^th^ day; and groups III to VII; oral dose of freeze-dried aqueous bark extract of *Nauclea orientalis* (L.) L. (0.125, 0.25, 0.5, 1.0, and 2.0 g/kg) for 2 weeks and single IP injection of Dox (18 mg/kg) on the 11^th^ day; IP injections were given after 16 h fasting in each animal. 72 h after the administration of IP injections, serum was collected from the blood for the detection of cardiac troponin I (cTnI), aspartate aminotransferase (AST, EC 2.6.1.1), and lactate dehydrogenase (LDH, EC 1.1.1.27), and myocardial tissues were fixed in 10% buffered formalin for histological assessment of myocardial damage.

### 2.6. Evaluation of Acute and Subchronic Toxicity of Plant Extract

Toxicity studies were followed on the optimum concentration of the aqueous bark extract which showed the very best cardio protection within the dose-response study. Both acute and chronic toxicity studies were performed following the principles of the Organization for Economic Cooperation and Development (OECD) [21, 22]. Two groups of Wistar rats were used in the acute toxicity study, and one group (control group) was administered with a single dose of water (10 mL/kg) orally. The second group was treated with a single dose (2.0 g/kg) of aqueous extract of *Nauclea orientalis* (L.) bark. Individual animals were inspected during the initial half an hour, then from time to time within the first 24 h, giving special attention during the initial 4 h. Then they were observed daily for 2 weeks by giving attention to changes in the mucous membranes, eyes, skin, fur, and general behaviours. Further, the signs of toxicity such as lethargy, salivation, diarrhoea, tremors, sleep, convulsions, and coma were also observed.

Healthy rats were randomly assigned into two groups in the subchronic toxicity study. The control group of rats was given an oral dose of D.H_2_O daily throughout one month, and the second group of rats received freeze-dried aqueous bark extract of *Nauclea orientalis* (L.) L. (2.0 g/kg) daily throughout one month via oral route. The experimental rats were inspected during the one-month period for the signs of toxicity and mortality. After 24 h of the last dose, the animals were sacrificed after 16 h fasting, and blood samples were collected into two tubes: one with EDTA for the assessment of haematological parameters including red blood cell (RBC) count, white blood cell (WBC) count, platelet count, haematocrit, haemoglobin concentration, mean corpuscular haemoglobin (MCH), mean corpuscular volume (MCV), and mean corpuscular haemoglobin concentration (MCHC) and the other without additives for the assessment of biochemical parameters including AST, alanine aminotransferase (ALT), alkaline phosphatase (ALP), creatinine, and blood urea. The weighed organs (heart, lungs, kidney, liver, small intestine, and spleen) were fixed in 10% buffered formalin for the detection of histologic evidences of toxicity.

### 2.7. Screening of *Nauclea orientalis* (L.) L. Aqueous Bark Extract for Cardioprotective Effect against Dox-Induced Oxidative Stress, Inflammation, and Apoptosis In Vivo

**Figure 1:**
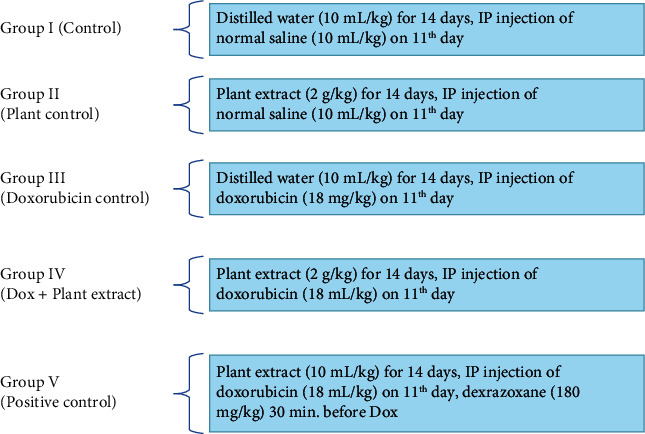
Treatment protocol.

Five groups (*n* = 10) of Wistar albino rats were subjected to the following test protocol. This study uses the method of Sandamali et al., and the method description partly reproduces their wording [23].  Figure 1 shows the summary procedure of the treatment protocol used in the study.

Group I received D.H_2_O by gastric gavage daily for 2 weeks, and an IP injection of normal saline was given on the 11^th^ day after a fasting period (16 h). Rats in group II were given a daily oral dose of freeze-dried aqueous bark extract (2.0 g/kg) for 2 weeks while an IP injection of normal saline was introduced on the 11^th^ day after a 16 h of fasting period. Group III and group V were also administered with an oral dose of D.H_2_O for 2 weeks, and in the 11^th^ day, both groups were injected with a single IP injection of Dox while the positive control group received single IP injection of dexrazoxane (180 mg/kg) half an hour before the introduction of Dox. Rats in group IV were given aqueous bark extract of *Nauclea orientalis* (2.0 g/kg) daily for 2 weeks, and a single respective dose of IP injection of Dox was administered after 16 h of fasting on the 11^th^ day.

At the end of the two weeks, all animals were sacrificed after 16 h of fasting, and blood samples were collected into tubes with no additives (plain tubes) for the determination of N terminal-pro-brain natriuretic peptide (NT-pro BNP), cTnI concentration, AST activity, and LDH activity, and the results were compared among the five groups utilized in the study.

The antioxidant parameters (glutathione peroxidase (GPx, EC 1.11.1.9), reduced glutathione (GSH), glutathione reductase (GR, EC 1.8.1.7), SOD (EC 1.15.1.1) activity, catalase (EC 1.11.1.6) activity, and total antioxidant level) and lipid peroxidation as an oxidative stress biomarker were determined using the homogenate (tissue weight to homogenization buffer: 1 : 10) of heart tissues prepared by half of the heart collected into ice-cold phosphate-buffered saline (PBS), and the results were compared among the five groups.

The rest of the heart tissues fixed in 10% buffered formalin were used in the determination of histological evidences of cardiac damage, in the immunohistochemical analysis of inflammatory and apoptotic markers, and in the terminal deoxynucleotidyl transferase-mediated dUTP nick-end labelling (TUNEL) assay.

The myeloperoxidase (MPO, EC 1.11.2.2) activity which was used as a biochemical inflammatory marker was measured in the serum of experimental rats.

#### 2.7.1. Collection of Blood (Cardiac Puncture) and Heart Tissues

Experimental Wistar rats were anesthetized using isoflurane inhalation and euthanized by cardiac exsanguination [24]. They were placed in the recumbent supine position on the animal operation table; an incision was made on the ventral aspect of the thoracic wall just above the diaphragm by using a surgical scissor, and the thoracic cavity was opened. Then the blood sample was collected slowly using a 21G needle from the ventricles. After that, the heart was collected from the mediastinum by dissecting it out from the major blood vessels. The heart was washed in saline, soaked in a blotting paper to extract the blood, and fixed in 10% buffered formalin. The myocardial tissue was sampled for histological assessment from the ventricle, 5 mm above the apex of the heart of all animals.

#### 2.7.2. Histological Assessment of Cardiac Damage

The heart tissues fixed in 10% buffered formalin were processed and embedded in paraffin. Then they were sectioned at 3 *μ*m thickness and stained with haematoxylin and eosin (H&E) [23]. The sections were examined by a histopathologist blinded to the treatments under the compound light microscope, and histological features of the necrosis were scored. The grading system was prepared by the authors of the study by inspecting the myocardium of rat tissues (section with 5 mm diameter) as follows: absence of any myocardial cells with features of necrosis: 0; ≤10 myocardial cells with features of necrosis: 1; 11-50 myocardial cells with features of necrosis: 2; 51-100 myocardial cells with features of necrosis: 3; and >100 myocardial cells with features of necrosis: 4.

Necrotic myocytes were identified with the features including hypereosinophilic cytoplasm, without striations and changes in the nucleus such as pyknosis, karyolysis, or karrheorhexis. Necrotic myocytes were counted separately in the subendocardial region and peripheral region of the rat myocardium.

#### 2.7.3. Immunohistochemical Analysis of Inflammatory Markers, TNF*α* and Apoptotic Markers, Caspase-3, and Bcl-2

Immunohistochemical analysis was followed on the basis of a modified method explained by Bulut et al. [25]. Antigen retrieval from the heart tissue sections was performed by placing them in a citrate buffer (pH 6.0). Then the sections were subjected to overnight incubation with the primary antibodies for TNF*α*, Bcl-2, and caspase-3 at 4°C. They were treated with the streptavidin peroxidase substrate solution, and then chromogen and diaminobenzidine (DAB) were applied followed by the counterstain haematoxylin. The breast carcinoma tissue (for TNF*α*), reactive lymph node tissue (for Bcl-2), and colon tissue (for caspase-3) were used as positive controls for immunostaining. The freely downloadable Image J 1.53a (2015) software [26] was applied to measure the area of expression of the respective markers which showed the brown colour staining on the tissue sections, and finally, the average area (in m^2^) of brown-stained area was calculated.

#### 2.7.4. TUNEL Assay

DNA fragmentation considered one of the features of apoptosis was detected using the TUNEL technique [27]. The DeadEnd™ Colorimetric TUNEL System detection kit (Promega Corporation, USA) was used, and the manufacturer's protocol was followed. In the presence of terminal deoxynucleotidyl transferase, 3′-OH ends of the fragmented DNA in apoptotic cells were labelled with biotinylated nucleotide. Then horseradish peroxidase-labelled streptavidin was bound to the biotinylated nucleotide and was detected using the hydrogen peroxide and stable chromogen, diaminobenzidine which gave the brown colour. Then the number of apoptotic nuclei in the 10 high-power field (×400 magnification) was counted.

### 2.8. Statistical Analysis

Data were expressed as the mean ± standard deviation (SD). For a statistical analysis of the data, the group means were compared by the one-way analysis of variance with post hoc analysis (SPSS 26.0 software). Dunnett's post hoc test was applied to identify significance among groups; *p* < 0.05 was considered statistically significant.

## 3. Results

### 3.1. Standardization of Plant Material

#### 3.1.1. Physicochemical and Phytochemical Analysis

The physicochemical analysis of *Nauclea orientalis* (L.) L. bark is shown in Supplementary data Table 1. The highest quantity of extractable matters was detected in hot water. Heavy metals including arsenic (As), cadmium (Cd), lead (Pb), and mercury (Hg) were absent in the *Nauclea orientalis* bark. The microscopic appearance of the bark is also included in Table 1 (Supplementary data).

The phytochemical profile of the *Nauclea orientalis* (L.) L. bark is shown in the Supplementary data Table 1. Polyphenols, tannins, alkaloids, and reducing sugars were detected in the bark. However, the *Nauclea* bark was negative for cyanogenic glycosides, anthracene glycosides, and cardenoloid glycosides. Saponin, flavonoids, and proteins were also not detected in the *Nauclea* bark.

### 3.2. *In Vitro* Antioxidant Activity of the *Nauclea orientalis* (L.) L. Bark

**Figure 2:**
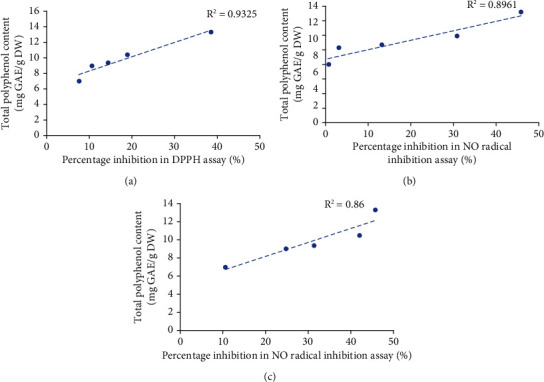
*In vitro* antioxidant activity as determined by the total polyphenol content of freeze-dried aqueous extract of *Nauclea orientalis* bark. (a) Correlation between polyphenol amount and the percentage inhibition in DPPH assay. (b) Correlation between polyphenol amount and the percentage inhibition in NO assay. (c) Correlation between polyphenol amount and the FRAP assay results. *R*^2^: correlation coefficient. DPPH: 2,2′-diphenyl-2-picrylhydrazyl hydrate. NO: nitric oxide. FRAP: ferric reducing antioxidant power.

The *ex vivo* antioxidant activity and polyphenol content of the aqueous extract of *Nauclea orientalis* are included in Table 2 (Supplementary data). The correlation between the polyphenol content and the in vitro antioxidant activities of aqueous plant extract was determined using the linear regression analysis, and results are shown in Figure 2. A considerable positive correlation was detected between the polyphenolic content and antioxidant activities (*R*^2^ = 0.9325, *R*^2^ = 0.8961, and *R*^2^ = 0.86, respectively). Therefore, it is evident that there is a substantial impact of phenolic substances to the antioxidant activity detected in the aqueous bark extract of *Nauclea orientalis*.

### 3.3. Dose-Response Effect of Aqueous Bark Extract of *Nauclea orientalis* (L.) L. for Cardioprotective Effect against Dox-Induced Cardiotoxicity In Vivo

**Figure 3:**
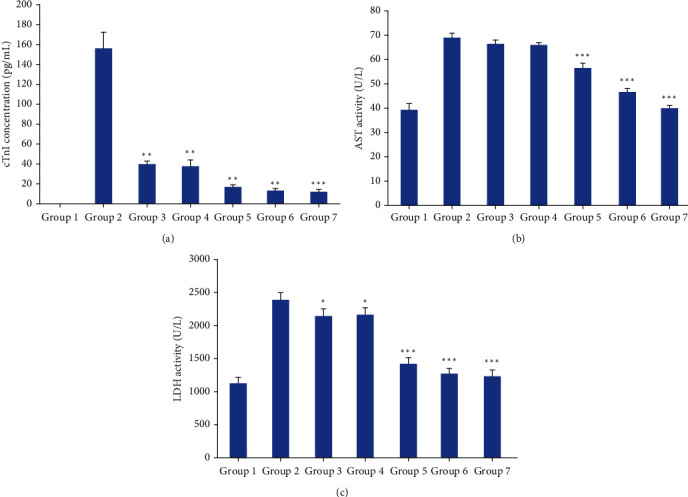
Biochemical investigation of the dose-response effect of freeze-dried aqueous *Nauclea orientalis* bark extract in Wistar rats treated with Dox. (a) Cardiac troponin I (cTnI) concentration in rats' serum. (b) Aspartate aminotransferase (AST) level in rats' serum. (c) Lactate dehydrogenase (LDH) level in rats' serum. Group 1: normal control, group 2: Dox control, group 3: rats treated with Dox (18 mg/kg) and *Nauclea* bark extract (0.125 g/kg), group 4: rats treated with Dox (18 mg/kg) and *Nauclea* bark extract (0.25 g/kg), group 5: rats treated with Dox (18 mg/kg) and *Nauclea* bark extract (0.50 g/kg), group 6: rats treated with Dox (18 mg/kg) and *Nauclea* bark extract (1.0 g/kg), and group 7: rats treated with Dox (18 mg/kg) and *Nauclea* bark extract (2.0 g/kg). Each column represents the mean ± SD (*n* = 10). ^∗^*p* < 0.05, ^∗∗^*p* < 0.01, ^∗∗∗^*p* < 0.001, significant difference compared to the Dox control.

Rats in the Dox control group showed a momentous rise (*p* < 0.001) in the serum cTnI level at 156.76 pg/mL in comparison to the control at 0 pg/mL (Figure 3(a)). After the administration of *Nauclea orientalis* bark extract, a considerable decrease (*p* < 0.05) in cTnI concentration was detected in all rat groups in comparison to the Dox control (groups III-VII: 41.04, 38.96, 17.75, 13.75, and 13.0 pg/mL). A significant increase (*p* < 0.001) in the enzyme activities of AST and LDH was also observed in rat groups administered with Dox in comparison to the normal control (Figures 3(b) and 3(c)). Although the rat groups treated with all concentrations of *Nauclea orientalis* bark extract showed significant changes (*p* < 0.05) against the Dox control group of rats for the LDH activity, a significant difference (*p* < 0.05) in the AST activity was detected only in rat groups treated with higher doses including 0.5, 1.0, and 2.0 g/kg.

**Figure 4:**
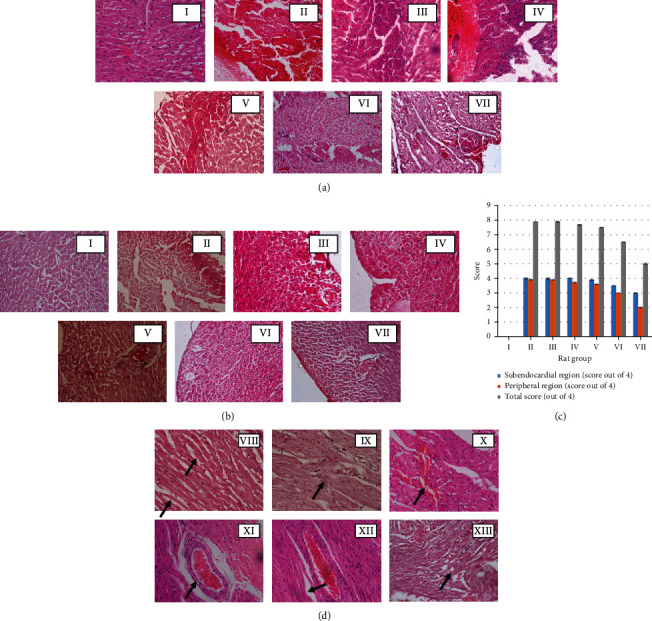
Histological investigation of the dose-response effect of freeze-dried aqueous *Nauclea orientalis* bark extract in Wistar rats treated with Dox (H&E, 10 × 40). (a) Cardiac myocytes with early changes of necrosis in the subendocardial region. (b) Cardiac myocytes with early changes of necrosis in the peripheral region. (c) Average grading of cardiac myocytes with early changes of necrosis (absence of any myocardial cells with features of necrosis: 0; ≤10 myocardial cells with features of necrosis: 1; 11-50 myocardial cells with features of necrosis: 2; 51-100 myocardial cells with features of necrosis: 3; and >100 myocardial cells with features of necrosis: 4). I: control, II: Dox control, III: rats administered with Dox (18 mg/kg) and *Nauclea* bark extract (0.125 g/kg), IV: rats administered with Dox (18 mg/kg) and *Nauclea* bark extract (0.25 g/kg), V: rats administered with Dox (18 mg/kg) and *Nauclea* bark extract (0.5 g/kg), VI: rats administered with Dox (18 mg/kg) and *Nauclea* bark extract (1.0 g/kg), and VII: rats administered with Dox (18 mg/kg) and *Nauclea* bark extract (2.0 g/kg). (d) Dox-induced reversible histologic changes in the myocardium of Wistar rats. VIII: interstitial oedema, IX: inflammatory infiltrations, X: haemorrhages, XI: congestion of blood vessel, XII: wavy myocardial fibers, and XIII: intracellular vacuoles. Arrows indicate reversible cellular changes. Each column represents the mean ± SD (*n* = 10).

Cross-section of the tissues of the myocardium of the normal control group exhibited normal morphology (Figures 4(a) and 4(b)). Rats administered with Dox alone exhibited the maximum score (7.9) for the early changes of necrosis showing greater damage to the peripheral and subendocardium of the myocardial tissues (Figures 4(a)–4(c)). All groups of rats subjected to the treatment of different doses of plant extract (group III-VII) showed a regular decrease in the score of myocardial cells with early changes of necrosis with the increase of the dosages of plant extract (Figure 4(c)). Additionally to the necrosis, other histological changes including congestion of blood vessels, intracellular vacuoles, interstitial oedema, haemorrhages, inflammatory infiltrations, and wavy myocardial fibers were also seen in the rats treated only with Dox (Supplementary data Table 3, Figure 4(d)). Groups of rats administered with different doses of plant extract exhibited different degrees of above histologic changes while haemorrhages, interstitial oedema, and inflammatory infiltrations were totally absent in groups of rats administered with higher dosages of the plant extract. Supporting the collective results obtained for biochemical parameters and histopathological assessment of cardiac damage, 2.0 g/kg of *Nauclea orientalis* bark extract was considered the optimum dosage to be used for the determination of cardioprotective activity against Dox-induced cardiotoxicity.

### 3.4. Acute and Subchronic Toxicity Evaluation of Plant Extract

The acute toxicity study revealed that rats treated with a single dose of *Nauclea* bark extract (2.0 g/kg) did not show mortality or morbidity throughout the 14-day period. No changes were observed in the morphology of the eyes, nose, fur, and skin. The respiratory and central nervous system effects such as tremors, convulsions, salivation, diarrhoea, and lethargy were also not examined in animals that were administered with a single dose of bark extract, and they did not show any unusual behaviour throughout the observation period.

Oral administration of freeze-dried aqueous extract of *Nauclea orientalis* bark daily for 30 days did not produce any conspicuous symptoms of toxicity in experimental Wistar rats. There was no treatment-related mortality reported in rats treated with the plant extract. No changes were observed in food and water intake during the period of investigation. Changes in body weight during the period of investigation are presented in Supplementary data, Table 4. Statistically significant changes (*p* > 0.05) in the gain of body weight were not detected between the control and the plant extract-treated group.

The subchronic administration of *Nauclea orientalis* bark extract on the changes in haematological parameters was presented in Supplementary data, Table 5. The measured haematology parameters were within the physiological range during the 30-day period of investigation, and a significant difference (*p* > 0.05) was not detected in comparison to the rats in the control group. Liver function (AST, ATL, and ALP activities) and kidney function (blood urea and creatinine) test results of the study groups are shown in Supplementary data, Table 6. Statistically considerable changes (*p* > 0.05) were not examined in those biochemical tests between the rats in the control group and the plant extract-administered group.

Absolute and relative organ weights of *Nauclea*-administered rats and control rats were shown in Supplementary data, Table 7. A significant change was not observed between the plant extract-treated group and the control group. No evidences of histological changes were examined in the six organs isolated (Supplementary data, Figure 1). The microscopic examination of the heart, kidneys, lungs, liver, small intestine, and spleen did not show any structural changes in the cells when viewed under the compound light microscope with multiple magnifications.

### 3.5. Screening of *Nauclea orientalis* (L.) L. Aqueous Bark Extract for Cardioprotective Effect against Dox-Induced Oxidative Stress, Inflammation, and Apoptosis In Vivo

#### 3.5.1. Cardiac Biomarkers in Serum

The intracellular cTnI is released into the circulation when there is damage to the cardiomyocytes. The rat group treated with Dox (Dox control) showed the maximum concentration of cTnI among the groups of rats utilized in this experiment indicating that Dox causes damage to the myocardium as shown in Table 1. Rats pretreated with *Nauclea orientalis* bark extract followed by Dox presented a substantial reduction (*p* < 0.05) in cTnI concentration in comparison to the Dox control. The rats in plant control group did not show significant change (*p* < 0.05) in cTnI concentration compared to the control group of rats. The rat group treated with *Nauclea* bark extract and Dox and the positive control group showed a significant change (*p* < 0.001) in cTnI concentration compared to the control group of rats.

NT-pro BNP is similarly measured as a suitable biomarker in the identification of cardiotoxicity. Considering the results, it could be noticed that NT-pro BNP concentration is significantly augmented (*p* < 0.05) in rats treated with Dox compared to the control group as exhibited in Table 1. Rats pretreated with aqueous *Nauclea* bark extract followed by Dox treatment exhibited a significantly low (*p* < 0.05) value in comparison to the rats in the Dox control group. The rats treated with *Nauclea* bark extract which was considered the plant control did not show any noteworthy change (*p* < 0.05) in the NT-pro BNP concentration related to the rats in the control group. However, the rat group treated with plant extract and Dox and the positive control group showed a significant increase (*p* < 0.001) in the NT-pro BNP concentration compared to the control.

AST and LDH are valuable cardiac enzymes which are released into the circulation when there is necrosis. The rats in the plant control group did not show any important difference (*p* < 0.05) in AST and LDH activities in relation to the rats in the control group as exhibited in Table 1. The AST and LDH activities were considerably elevated (*p* < 0.05) in Dox-administered rats in comparison to the rats in the control group. The rats in the plant extract-pretreated group followed by Dox injection exhibited a significant decrease (*p* < 0.05) in AST and LDH activities in comparison to the rats in the Dox control group. Although the rats in group IV (*Nauclea* + Dox) showed a significant elevation (*p* < 0.05) in AST and LDH activities compared to the control, the positive control group did not show a significant elevation (*p* > 0.05) compared to the control.

#### 3.5.2. Antioxidant Parameters in Homogenate of Heart Tissue

**Figure 5:**
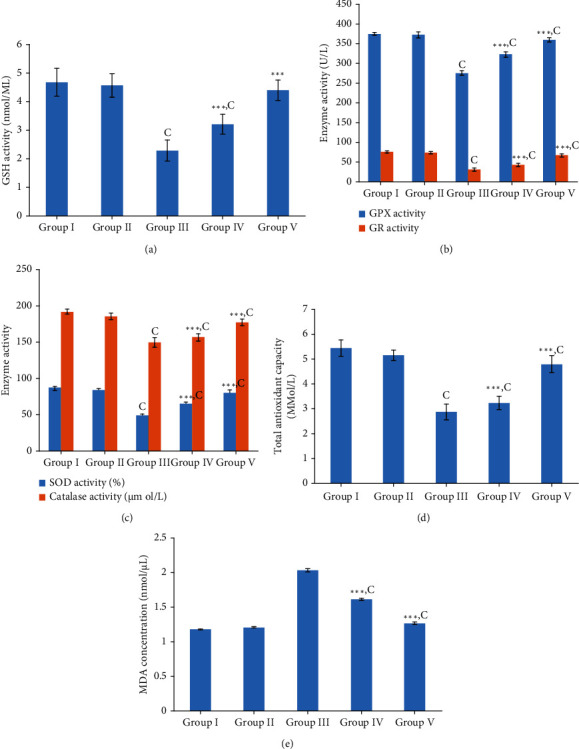
Investigation of oxidative stress biomarkers in the screening of aqueous extract of *Nauclea orientalis* (L.) L. bark for the cardioprotective effect. (a) Effect of plant extract on GSH activity of homogenate heart tissues. (b) Effect of plant extract on GPx and GR activity of homogenate heart tissues. (c) Effect of plant extract on SOD and catalase activity of homogenate heart tissues. (d) Effect of plant extract on total antioxidant activity of homogenate heart tissues. (f) Effect of plant extract on lipid peroxidation in the homogenate heart tissues. Group I: control group, group II: *Nauclea orientalis* plant control, group III: Dox control, group IV: rats received *Nauclea orientalis* bark extract + Dox, and group V: positive control (rats received dexrazoxane + Dox). *p* values ^∗^<0.05, ^∗∗^<0.01, and ^∗∗∗^<0.001 were considered significant (compared to the Dox control); *p* values ^a^<0.05, ^b^< 0.01, and ^c^<0.001 were considered significant (compared to the control). Each column represents the mean ± SD (*n* = 10). GSH: reduced glutathione, GPx: glutathione peroxidase, GR: glutathione reductase, SOD: superoxide dismutase, and MDA: malondialdehyde (end product of lipid peroxidation).

The results of GSH, GPx, and GR activities among the groups of rats utilized in this experiment are shown in Figures 5(a) and 5(b). The glutathione system has a key role in protecting the cells against oxidative damage. Dox induces damage to the myocardium due to oxidative stress generated by ROS, and this was reinforced by the results found in the present study as well revealing a considerable reduction (*p* < 0.001) in GSH, GPx, and GR enzyme activities in myocardial tissues of Wistar rats subjected to the treatment of Dox and D.H_2_O compared to the rats of the control group. However, the rats in the pretreated plant extract group followed by the Dox injection showed a significant (*p* < 0.05) elevation in the GSH level as well as other two antioxidant enzyme (GPx and GR) activities in relation to the rats in the Dox control group. Rats in the plant extract control group did not exhibit any substantial difference (*p* > 0.05) in the GSH level and other two enzyme activities in comparison to the rats in the control. However, the rats in group IV (*Nauclea* + Dox) showed a significant reduction (*p* < 0.05) in GSH, GR, and GPx activities while the positive control showed a significant reduction (*p* < 0.05) only in GPx and GR activities.

In the present study, antioxidant enzymes which detoxify oxygen radicals like catalase and SOD also displayed a significant rise (*p* < 0.05) in the rats in the Dox control group in comparison to the rats in the normal control group as shown in Figure 5(c) representing free radical formation following Dox treatment. However, rats pretreated with freeze-dried aqueous extract of *Nauclea orientalis* bark followed by Dox injection were capable of significantly augmenting (*p* < 0.05) the SOD and catalase enzyme levels in comparison to the rats in the Dox control group. Considerable differences (*p* > 0.05) could not be detected in these two enzyme activities in the heart tissues of rats treated with aqueous *Nauclea* bark extract alone (plant control) when compared to the control group of rats. The positive control group and rat group treated with plant extract and Dox showed significant reductions (*p* < 0.05) in SOD and catalase activities compared to the control.

The total antioxidant status assessed among the groups of experimental rats is shown in Figure 5(d). Wistar rats in the Dox control group exhibited the significant drop (*p* < 0.001) in total antioxidant status when compared with the control group of rats confirming that Dox causes the exhaustion of antioxidants within the myocardial tissues. However, Wistar rats pretreated with the *Nauclea* bark extract followed with Dox injection were capable of significantly increasing (*p* < 0.001) the total antioxidant status in comparison to the Wistar rats in Dox control indicating its high antioxidant effect. The plant extract control did not show a significant difference (*p* > 0.05) in total antioxidant activity compared to the control. However, rats treated with *Nauclea* bark extract and Dox showed a significant reduction (*p* < 0.001) in total antioxidant activity compared to the control.

#### 3.5.3. Analysis of Lipid Peroxidation in Homogenate of Heart Tissue

Results of lipid peroxidation by means of malondialdehyde (MDA) concentration are presented in Figure 5(e). The *Nauclea* bark extract control group did not exhibit any considerable difference (*p* > 0.05) in the lipid peroxidation compared to the normal control. Rats in the Dox control exhibited a significant rise (*p* < 0.05) in lipid peroxidation in comparison to the rats in the control group indicating that Dox treatment increases the lipid peroxidation. Although the rats in the group pretreated with freeze-dried aqueous plant extract followed by Dox treatment showed a significant increase (*p* < 0.001) in lipid peroxidation compared to the control, they exhibited a substantial decrease (*p* < 0.001) in the MDA level in comparison to the rats in the Dox control group.

#### 3.5.4. MPO Activity in Serum

**Figure 6:**
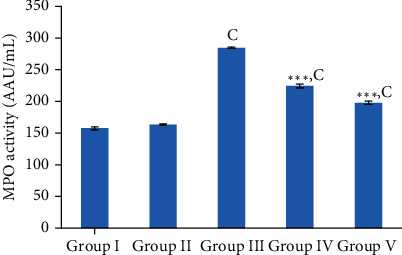
Investigation of serum inflammatory marker (MPO activity) in the screening of aqueous extract of *Nauclea orientalis* (L.) L. bark for the cardioprotective effect. Group I: control group, group II: *Nauclea orientalis* plant control, group III: Dox control, group IV: rats received *Nauclea orientalis* bark extract + Dox, and group V: positive control (rats received dexrazoxane + Dox). *p* values ^∗^<0.05, ^∗∗^<0.01, and ^∗∗∗^<0.001 were considered significant (compared to the Dox control); *p* values ^a^<0.05, ^b^< 0.01, and ^c^<0.001 were considered significant (compared to the control). Each column represents the mean ± SD (*n* = 10). MPO: myeloperoxidase.

Inflammation is another obvious outcome of the Dox treatment which is evident by increased MPO activity. The results of the MPO activity among the experimental groups of the present study are shown in Figure 6. The plant extract control group did not show a considerable difference (*p* > 0.05) in MPO activity when compared with the control group. Wistar rats in the Dox control group showed a significant rise (*p* < 0.001) in MPO activity in comparison to the rats in the control group, and it was the highest concentration among the five groups of rats. However, rats in the group pretreated with the plant extract followed by Dox treatment showed a considerable reduction (*p* < 0.001) in the MPO activity compared to the rats in the Dox control group.

Rats in the positive control group were treated with dexrazoxane as it is the accepted protective agent in the clinical setting to be used to treat the Dox-induced cardiotoxicity. When considering the biochemical results of the present experiment, a significant difference (*p* < 0.001) was observed between the rats in the positive control group and the Dox control in all parameters tested.

#### 3.5.5. Histological Assessment of the Myocardial Damage

**Figure 7:**
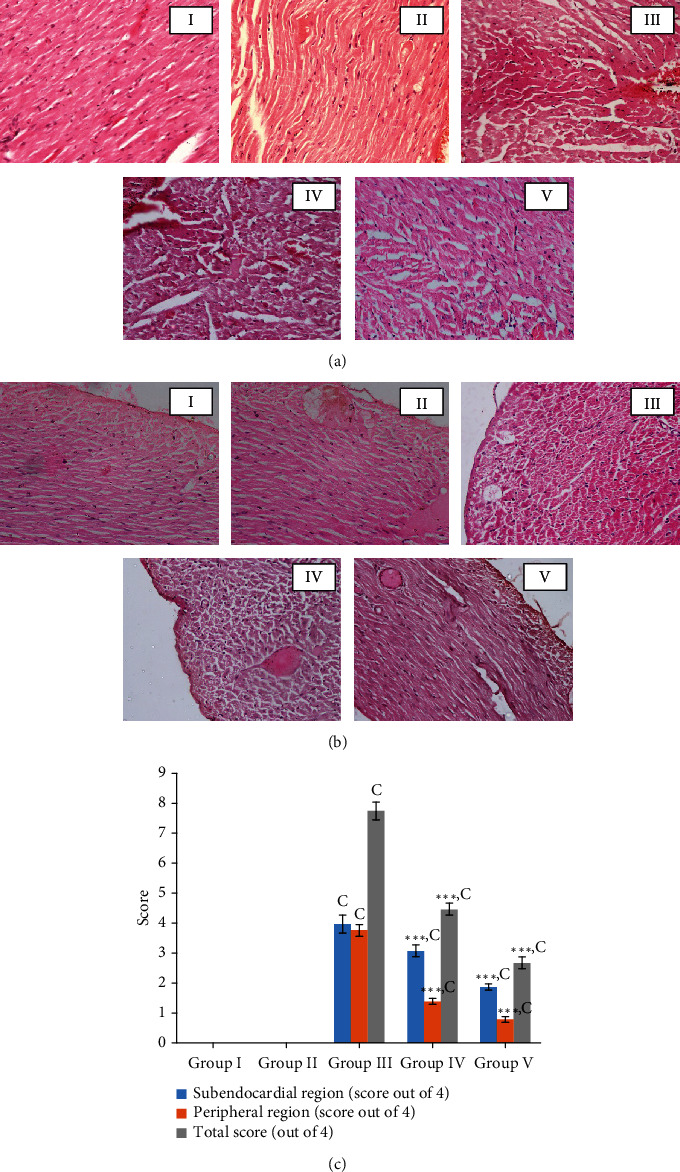
Histological investigation in the screening of aqueous extract of *Nauclea orientalis* (L.) L. bark for the cardioprotective effect. (a) Cardiac myocytes with early changes of necrosis in the subendocardial region (H&E, 10 × 40). (b) Cardiac myocytes with early changes of necrosis in the peripheral region (H&E, 10 × 40). (c) Average grading of cardiac myocytes with necrotic changes (absence of any myocardial cells with features of necrosis: 0; ≤10 myocardial cells with features of necrosis: 1; 11-50 myocardial cells with features of necrosis: 2; 51-100 myocardial cells with features of necrosis: 3; and >100 myocardial cells with features of necrosis: 4). Group I: control group, group II: *Nauclea orientalis* plant control, group III: Dox control, group IV: rats received *Nauclea orientalis* bark extract + Dox, and group V: positive control (rats received dexrazoxane + Dox). *p* values ^∗^<0.05, ^∗∗^<0.01, and ^∗∗∗^<0.001 were considered significant (compared to the Dox control); *p* values ^a^<0.05, ^b^< 0.01, and ^c^<0.001 were considered significant (compared to the control). Each column represents the mean ± SD (*n* = 10).

The myocardial tissues of the rats in the control group exhibited the general architecture in the peripheral and subendocardial regions of the myocardium (Figures 7(a) and 7(b)). A large number of cells with early changes of necrosis were observed in both regions (peripheral and subendocardium) of the myocardium of the Dox control group of rats, and they exhibited the highest score (7.8) among the five groups of rats used in the present study (Figure 7(c)). However, a number of cells with necrotic changes were more visible in the subendocardial region when compared with the peripheral region (Figures 7(a) and 7(b)). Features of cell injury including intracellular vacuoles, congestion of blood vessels, interstitial oedema, haemorrhages, and wavy myocardial fibers (Supplementary data Table 8, Figure 4(d)) were observed in rats of the Dox control group. Pretreatment with the freeze-dried *Nauclea orientalis* bark extract reduced the early changes of necrosis as evident by the lesser number of necrotic cells being visible with a score of 4.5 (Figure 7(c)). However, cells with early changes of necrosis were more noticeable in the subendocardial region. Only a few reversible changes of cell injury including occasional intracellular vacuoles, congestion of blood vessels, and wavy myocardial fibers were observed while interstitial oedema, inflammatory cell infiltrations, and haemorrhages were absent in the rat group which underwent pretreatment with plant extract followed by Dox treatment (Supplementary data Table 8). The plant extract control group (group II) did not show any histological changes in the myocardium and were the same as the control group (Figures 7(a) and 7(b)). The Wistar rats in the positive control group showed well-preserved myocardium with a minimum score in the necrosis scale, and the intracellular vacuoles were observed as the only reversible histological changes of cell injury.

#### 3.5.6. Immunohistochemical Analysis of Inflammatory Markers (TNF*α*) and Apoptotic Markers (Caspase-3 and Bcl-2)

**Figure 8:**
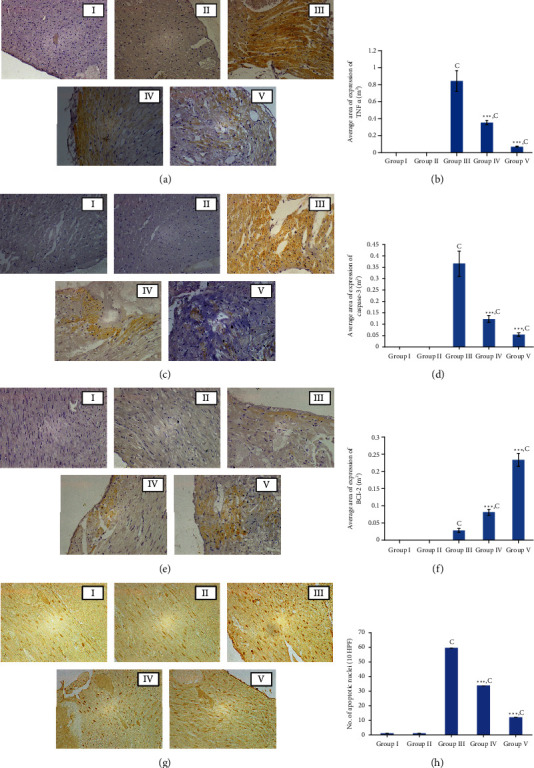
Immunohistochemical analysis of inflammatory markers (TNF*α*) and apoptotic markers (caspase-3 and Bcl-2) and TUNEL assay. (a) Microscopic observation of immunohistochemical analysis of TNF*α* (10 × 40). (b) Average area of expression of TNF*α* in myocardial tissues of rats. (c) Microscopic observation of immunohistochemical analysis of caspase-3 (10 × 40). (d) Average area of expression of caspase-3 in myocardial tissues of rats. (e) Microscopic observation of immunohistochemical analysis of Bcl-2 (10 × 40). (f) Average area of expression of Bcl-2 in myocardial tissues of rats. (g) Microscopic analysis of TUNEL-positive nuclei (10 × 40). (h) Average number of TUNEL-positive nuclei in myocardial tissues of rats. Group I: control group, group II: *Nauclea orientalis* plant control, group III: Dox control, group IV: rats received *Nauclea orientalis* bark extract + Dox, and group V: positive control (rats received dexrazoxane + Dox). *p* values ^∗^<0.05, ^∗∗^<0.01, and ^∗∗∗^<0.001 were considered significant (compared to the Dox control); *p* values ^a^<0.05, ^b^<0.01, and ^c^<0.001 were considered significant (compared to the control). Each column represents the mean ± SD (*n* = 10). TNF*α*: tumour necrosis factor *α*, Bcl-2: B-cell lymphoma 2, and TUNEL: terminal deoxynucleotidyl transferase dUTP nick-end labelling.

Expression of respective inflammatory and apoptotic markers was visualized in brown colour in immunostained cardiomyocytes. The microscopic appearance of expression of TNF*α* is shown in Figure 8(a). Expression of TNF*α* which is an inflammatory marker was absent in the control group of rats and rats treated with *Nauclea orientalis* bark extract (plant control) alone. However, the expression of TNF*α* was markedly elevated in rats administered with Dox, and they showed the highest amount of cytokine expression with an average area of expression of 0.85 m^2^ among the experimental groups as shown in Figure 8(b). Pretreatment with lyophilized aqueous *Nauclea orientalis* bark extract in rats treated with Dox showed a considerable (*p* < 0.001) decrease in the expression of TNF*α* when compared with the Dox control as evident in the reduction of area which expresses the TNF*α* (Figure 8(b)). Dexrazoxane-treated rats (positive control group) also exhibited a significant decrease (*p* < 0.001) in the expression of TNF*α* in comparison with the Dox control group.

Caspase-3 is a key protein marker expressed in apoptosis. Microscopic evidence of expression of caspase-3 is shown in Figure 8(c). Expression of caspase-3 was not observed in the control group of rats and rats treated with the plant extract alone. Treatment with Dox (Dox control) markedly increased the level of caspase-3 as shown in Figure 8(d), and this group of rats showed the highest expression of caspase-3 among the study groups which was evident by the highest area (0.37 m^2^) of expression. Interestingly, pretreatment with aqueous bark extract of *Nauclea orientalis* significantly reduced (*p* < 0.001) the area of expression of caspase-3 protein compared to the Dox control group as shown in Figure 8(d). The positive control group that underwent dexrazoxane treatment showed the lowest level of caspase-3 which was evident by the lowest area of expression.

Bcl-2 is considered an antiapoptotic marker.  Figure 8(e) shows the microscopic observations of Bcl-2 expression. Expression of Bcl-2 marker was absent in the control group as well as in the rat group treated only with the aqueous plant extract. A markedly increased level of Bcl-2 marker (average area of expression was 0.24 m^2^) was observed in the positive control group of rats that were treated with dexrazoxane. On the other hand, the lowest expression of antiapoptotic marker, Bcl-2, was observed in rats treated with Dox alone (Dox control) which was evident with the lowest area (0.03 m^2^) of expression. However, a significant increase (*p* < 0.001) in Bcl-2 expression was observed in rats pretreated with aqueous bark extract of *Nauclea orientalis* that were exposed to Dox injection compared to the Dox control group as shown in Figure 8(f).

#### 3.5.7. TUNEL Assay

TUNEL assay detects DNA cleavage which is considered an early nuclear change of apoptosis. The microscopic appearance of the TUNEL stained myocardium is shown in Figure 8(g). Some rats in the control group as well as the plant extract control group showed very few numbers of TUNEL-positive nuclei, and the results are shown in Figure 8(h). The Dox control group of rats showed significantly (*p* < 0.001) increased the number of TUNEL-positive nuclei compared to the control group, and the average number of TUNEL-positive nuclei per 10 high-power fields was 59.6. However, pretreatment with lyophilized aqueous bark extract of *Nauclea orientalis* in rats treated with Dox showed a significant reduction (*p* < 0.01) in apoptotic nuclei compared to the Dox control group, and the average number of apoptotic nuclei in 10 high-power fields was 33.8. The positive control group of rats treated with dexrazoxane also showed a significant reduction (*p* < 0.001) in the number of apoptotic nuclei compared to the Dox control group.

## 4. Discussion

Dox, the most potent anthracycline chemotherapeutic agent, exhibits acute and chronic cardiotoxicity which leads to left ventricular dysfunction and subsequent heart failure [28]. A solid body of evidence suggests that oxidative stress, inflammation, and apoptosis are main mechanisms involved in the pathogenesis of Dox-induced cardiotoxicity [29].

The molecular mechanism of cardiotoxicity is multifactorial, and the most accepted mechanism is the generation of ROS related to oxidative stress [5]. Due to the importance of oxidative stress in the occurrence of Dox-induced cardiotoxicity, strategies that are capable of reducing oxidative stress have been identified as effective approaches to prevent cardiotoxicity [7]. Based on this, combination therapies of drugs with antioxidants have been investigated in many studies. Therefore, in the present study, a medicinal plant which showed a higher antioxidant activity was selected to be screened against Dox-induced cardiotoxicity.

Although oxidative stress plays a major role in the pathogenesis of Dox-induced cardiotoxicity, Dox-induced inflammatory effects on the myocardium and the vasculature are mediated through the upregulation of NF-*κ*B expression [30]. Furthermore, there is accumulated evidence to suggest that mechanisms of programmed cell death, such as apoptosis which is characterized by nuclear and chromosomal DNA fragmentation, cell shrinkage, and blebbing as well as autophagy, also play an important role in the pathogenesis of cardiotoxicity [30, 31]. Therefore, assessment of markers for cardiac damage, oxidative stress, inflammation, apoptosis, and DNA fragmentation is important in the diagnosis of anthracycline-induced cardiotoxicity as well as in the determination of mechanism of action of therapeutic interventions used against them. In this study, we determined the effect of the aqueous bark extract of *Nauclea orientalis* on the oxidative stress, inflammation, apoptosis, and DNA fragmentation induced by the administration of Dox in Wistar rats.

According to the recommendations given in OECD guidelines to perform limit tests, a higher dose of 2.0 g/kg must be used to investigate the acute and subchronic toxicity of plant extracts [22, 23]. Previous studies have revealed that severe growth depression may occur due to reduced food intake which is a common phenomenon if plant extracts are toxic to the experimental animals [32]. In this study, in addition to the absence of any signs of acute toxicity, the consumption of food and intake of water were not changed between the control and treatment groups suggesting that this plant extract neither induced nor suppressed appetite in healthy rats. Further, a significant difference was not observed in the body weights of the test group compared to the control group. The analysis of biochemical and haematological parameters is also relevant for the evaluation of risk as they are widely used as predictors of toxicity of medicinal plant extracts to humans and animals [32]. The rat group treated with *Nauclea orientalis* bark extract did not show any significant changes in haematological parameters, liver function, and kidney function parameters suggesting that subchronic administration of plant extract had no toxic effects in Wistar rats. The histological assessment of the body tissues is considered the gold standard for the evaluation of treatment-related pathological changes in different tissues [33]. The microscopic examination of organs including the heart, kidneys, lungs, liver, small intestine, and spleen did not show any alterations in cell structure or any unfavourable effects when viewed under the light microscope using multiple magnifications confirming the nontoxic effect of *Nauclea orientalis* bark extract.

It is already proven that oxidative stress is the major cause of cardiotoxicity, and cardiomyocytes are more prone to this damage if their antioxidant defence mechanism is compromised [27, 34, 35]. Further, it is proven that supplementation of an exogenous antioxidant provides protection against cardiac injury. Therefore, *Nauclea orientalis* bark extract was screened for the potential cardioprotective effects against Dox-induced cardiotoxicity *in vivo*. Cardiotoxicity induced by Dox is manifested by increased activities of cTnI, NT-pro BNP, AST, and LDH which are released from damaged cardiomyocytes in response to the degree of cardiac injury, ischaemia, and infarction [34]. In the present study, it was revealed that *Nauclea orientalis* bark extract has the potential to significantly reduce the cardiac biomarkers increased in response to Dox administration suggesting that it is an effective approach to attenuate Dox-induced cardiotoxicity. Consistent with our results, a study done by Sergazy et al. has shown that Dox causes increased activity of AST and cardiac troponin T, and administration of grape polyphenol concentrate significantly reduced the release of these two parameters [36]. Another study done by El-Sayed et al. has shown that serum creatine kinase-MB activity and LDH activity increased with Dox treatment in rats, and *Curcuma longa* L. extract which has an antioxidant effect had the ability to significantly reduce the two cardiac biomarkers [37]. Afsar et al. also corroborated our results showing that *Acacia hydaspica* R. Parker significantly reduced the AST, LDH, and creatine kinase-MB activities which were increased by Dox treatment [34].

Elevated level of lipid peroxidation and alterations in the enzymatic and nonenzymatic antioxidant systems are considered the signs of oxidative stress induced by Dox [38]. GSH, GPx, GR, SOD, and catalase are the common antioxidant compounds found in tissues which protect tissues from oxidative stress injury. GSH is the most abundant intracellular antioxidant molecule, and the deficiency of GSH observed in the Dox-treated group could be due to the consumption of GSH during the interaction between Dox-induced free radicals with biological membranes, macromolecules, and subsequent lipid peroxidation [39]. In the present study, oxidative damage was observed in the Dox control group as shown by the markedly elevated MDA concentration and reduced GSH, GPx, GR, catalase, and SOD levels, but the aqueous *Nauclea* bark extract could significantly increase all antioxidant enzyme activities in plant extract-treated group suggesting its high antioxidant activity. According to the results of the present study, important phytochemicals such as polyphenols, alkaloids, and tannins were present in the *Nauclea orientalis* bark while toxic phytochemicals were absent. It was already reported that plants with high polyphenol content have significant antioxidant activity [40]. Results of this study also revealed that the *Nauclea orientalis* bark has a considerable polyphenol content which may have contributed to its antioxidant activity. A previous study conducted in Sri Lanka also reported that the aqueous extract of *Nauclea orientalis* bark extract possesses high antioxidant activity which corroborates the results obtained in our study [33]. A study done by Gnanapragasam et al. also confirmed this mechanism showing that *Centella asiatica* which has high antioxidant activity has the ability to significantly increase GSH and GPx activities in heart tissues after the administration of Dox [41]. SOD and catalase are also important antioxidant enzymes in the first-line defence mechanism which protect the biological systems from oxidative stress damages [42]. Dox treatment causes depletion of these enzyme activities as it produces many oxygen radicals [40]. A study done by Liu et al. reported that *Panax notoginseng* has a significant effect on the increase of SOD and catalase activities in mice injected with Dox [43]. Hence, results reported in many other studies are consistent with that of the present study as they have also shown that natural compounds with high antioxidant activities have the potential to attenuate Dox-induced cardiotoxicity [27, 35–38]. The formation of ROS in Dox metabolism can cause lipid peroxidation of the cell membrane which can increase the MDA concentration in the myocardial tissues [44]. A study done by Singh et al. also showed that administration of Dox causes significant increase in MDA concentration, and the aqueous extract of *Terminalia arjuna* bark which has significant antioxidant effect greatly reduced the lipid peroxidation which was evident by the low MDA concentration reported [35]. Several other studies have also confirmed the results reported by Singh et al. [27, 36–38]. The results of the present study were also in line with the above test results showing that *Nauclea orientalis* bark extract also has a significant capacity to reduce MDA production subsequent to Dox administration and could be an effective approach to reduce cardiotoxicity induced by Dox.

Histopathological evidence of cellular necrosis is considered the gold standard in the identification of Dox-induced cardiotoxicity in rodents [3, 45]. In the present study, the aqueous extract of the *Nauclea orientalis* bark significantly decreased the histological changes such as necrosis, intracellular vacuoles, congestion of blood vessels, and wavy myocardial fibers while histological changes including haemorrhages, oedema, and inflammatory infiltrations were completely absent in the plant-treated group indicating its cardioprotective activity. Consistent with our findings, a previous study done by Sun et al. also showed that myocardial fibrosis and necrosis are evident after the administration of Dox [46]. Further, Sun et al. showed that scutellarin, which has an antioxidant effect, significantly reduced the histological changes of myocardial damage [46]. Another study done by Zang et al. also showed that an antioxidant compound, oxymatrine, effectively reduced the histological changes such as necrosis, intracellular oedema, damaged mitochondria, and wavy cardiac fibers in Dox-treated rats [47]. Iqbal et al. reported that telmisartan attenuated the histological changes including focal necrosis, oedema, haemorrhage, and congestions in rat heart induced by a single dose of Dox [48].

Dox-induced cardiotoxicity is a multimolecular mechanism which is mainly led by oxidative stress, inflammation, and apoptosis. Previous studies reported that Dox-induced cardiotoxicity is initiated by the production of ROS. As Dox enters the body, it binds tightly to cardiolipin which is located in the inner mitochondrial membrane and accumulates in mitochondria. This affects the synthesis of ROS and reactive nitrogen species via the electron transport chain which subsequently cause mitochondrial and cellular membrane damage and diminished antioxidant defence system leading to cellular apoptosis [49]. Mitochondrial damage also initiates an imbalance in the intracellular Ca^2+^ concentration, which further affects the apoptosis pathways causing myocardial cell death [50]. Because of this molecular mechanism, it is expected that the use of plant extract which has a high antioxidant effect is capable of attenuating the apoptosis in cardiac tissues without obstructing the anticancer effect of Dox mainly led by DNA intercalation [5].

Dox-induced myocytic necrosis evokes an inflammatory response in the cardiac tissues by upregulating the expression of NF-*κ*B signalling which increases the secretion of inflammatory cytokines including TNF*α*, interleukin 1*β*, and interleukins 6 and 8. These cytokines subsequently cause profound pathological alterations which lead to cardiomyopathy [29]. Therefore, to investigate some of these mechanisms further, inflammatory markers were analysed where MPO activity was estimated in the serum, and the immunohistochemical analysis of TNF*α* was performed in the myocardial tissues. The results of the present study showed a significant reduction in the serum inflammatory marker, MPO, and expression of TNF*α* in myocardial tissues suggesting that *Nauclea orientalis* bark may also have significant anti-inflammatory activities. Consistent with the results obtained in this study, the study done by Zhang et al. showed that Dox treatment increases the inflammatory mediators including TNF*α* and interleukin 6 (IL-6) in the serum of mice, and injection of “shenmai” which is composed of *Panax ginseng* and *Ophiopogon japonicus* (Thunb.) suppressed the expression of TNF*α* and IL-6 [50]. Other studies done by Hijazi et al. and Hamza et al. also showed similar results [51, 52]. Programmed cell death, apoptosis, also plays a major role in Dox-induced cardiotoxicity which increases the cytochrome c, Bax expression, and caspase-3 activity [49]. Dox reduces the expression of Bcl-2, a major antiapoptotic protein which inhibits apoptosis for the protection of structure and function of mitochondria. In the present study, the TUNEL assay was performed to detect DNA fragmentation that results in apoptosis, and immunohistochemical markers for caspase-3 and Bcl-2 were used to identify involvement of apoptosis in Dox-induced cardiotoxicity. The TUNEL assay identifies DNA fragments which result in apoptosis [46]. Although DNA fragmentation is not specific to apoptosis as it occurs in necrosis as well, the use of TUNEL assay together with the upregulation of caspase-3 and downregulation of Bcl-2 which are known markers of apoptosis provides evidence for the presence of apoptosis [53]. In the present study, the Dox control group showed a marked elevation of TUNEL-positive nuclei, and the expression of caspase-3 and reduction in Bcl-2 expression compared to the control group confirmed the presence of apoptosis in Dox-treated rats. Pretreatment with aqueous extract of *Nauclea orientalis* bark showed a significant reduction in caspase-3 activity and TUNEL-positive nuclei as well as an elevation in expression of Bcl-2 marker showing that *Nauclea* bark may also have an antiapoptotic effect. Previous study done by Zhang et al. corroborated the results obtained in our study and has shown that luteolin which is a flavonoid found in vegetables and fruits attenuates the Dox-induced cardiotoxicity by upregulating the AKT/Bcl-2 pathway [54]. Several other studies also showed that natural compounds with antioxidant and antiapoptotic effects have the ability to attenuate Dox-induced cardiotoxicity [27, 55–58].

In the present study, dexrazoxane was used as the positive control as it is the only cardioprotective drug approved by the FDA for the prevention of anthracycline-induced cardiotoxicity [59]. However, when the results of the rat group treated with the plant extract were compared with the positive control, a significant difference (*p* < 0.05) was observed. This may be due to the differences in the mechanisms of action triggered by the active constituents in the plant extract and dexrazoxane to attenuate Dox-induced cardiotoxicity. Although the mechanism of dexrazoxane is not completely understood, numerous studies have proven that it is due to iron chelation which decreases the generation of ROS [60, 61]. But, in the present study, we assume that the cardioprotective effect of *Nauclea orientalis* bark is due to the replenishment of cardiomyocytes with antioxidants provided by the plant.

In the present study, cardiac biomarkers were analysed to detect the structural abnormalities in the heart of Wistar rats. However, cardiac function tests such as ECG analysis and echocardiography analysis could not be performed due to limited resources, and it is considered a limitation of this study.

**Figure 9:**
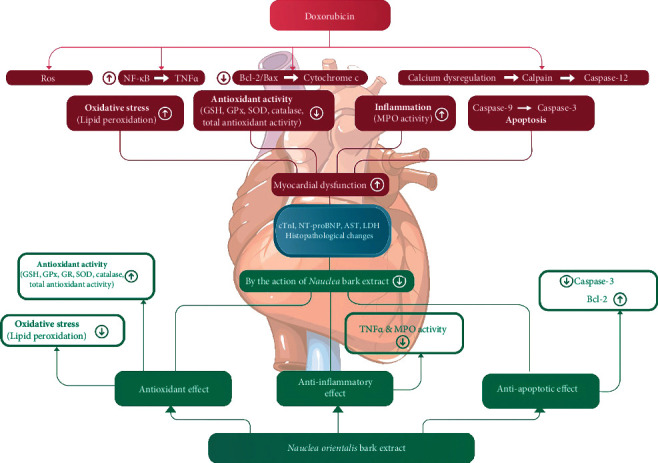
Schematic representation of cardioprotective effect of *Nauclea orientalis* bark extract.

In summary, as shown in Figure 9, formation of ROS during Dox metabolism induces the oxidative stress and also upregulates the inflammatory and apoptotic pathways in myocardial tissues. However, *Nauclea orientalis* bark extract which has high antioxidant activity significantly reduced the oxidative stress in the cardiac tissues. The current study also provided evidence that the *Nauclea orientalis* bark extract protected the cardiomyocytes of Wistar rats against Dox-induced cardiotoxicity via a synergistic effect through the suppression of oxidative stress, inflammation, and apoptosis.

## 5. Conclusion

In conclusion, administration of standardized aqueous bark extract of *Nauclea orientalis* (2.0 g/kg) attenuated the Dox-induced oxidative stress, inflammation, apoptosis, and DNA fragmentation, and it has the potential to be developed as an adjunct against Dox-induced cardiotoxicity in cancer patients who undergo anthracycline chemotherapy.

## Figures and Tables

**Table 1:** Investigation of cardiac biomarkers in the screening of aqueous extract of *Nauclea orientalis* (L.) L. bark for the potential cardioprotective effect.

Serum cardiac biomarkers	Group I	Group II	Group III	Group IV	Group V
cTnI (pg/mL)	0.00	0.00	145.15 ± 10.77^c^	38.92 ± 3.08^∗∗∗,c^	11.46 ± 2.59^∗∗∗,c^
NT-pro BNP (pg/mL)	41.57 ± 7.29	44.43 ± 4.27	371.14 ± 9.69^c^	233.29 ± 11.12^∗∗∗,c^	159.43 ± 12.39^∗∗∗,c^
AST (U/L)	25.71 ± 1.41	25.12 ± 1.74	66.10 ± 2.07^c^	34.98 ± 2.14^∗∗∗,c^	26.90 ± 1.26^∗∗∗^
LDH (U/L)	1057.21 ± 38.6	1181.06 ± 36.30	1584.19 ± 83.4^c^	1308.96 ± 68.8^∗∗,c^	1104.97 ± 58.7^∗∗∗^

cTnI: cardiac troponin I, NT-pro BNP: N terminal-pro-brain natriuretic peptide, AST: aspartate aminotransferase, LDH: lactate dehydrogenase. Group I: control group, group II: *Nauclea orientalis* plant control, group III: Dox control, group IV: rats received *Nauclea orientalis* bark extract + Dox, and group V: positive control. *p* values ^∗^<0.05, ^∗∗^<0.01, and ^∗∗∗^<0.001 were considered significant (compared to the Dox control). *p* values ^a^<0.05, ^b^<0.01, and ^c^<0.001 were considered significant (compared to the control).

## Data Availability

The datasets used and/or analysed during the current study are available from the corresponding author on reasonable request.

## Supplementary Materials

Supplementary Materials Table S1: physicochemical properties and phytochemical analysis of *Nauclea orientalis* bark. Table S2: total polyphenol content and the in vitro antioxidant activity of aqueous bark extract of *Nauclea orientalis* (L.) L. bark. Table S3: dose-response effect on reversible histological changes of cardiac tissues of Wistar rats exposed to different doses of *Nauclea orientalis* bark extracts. Table S4: effect of subchronic oral administration of *Nauclea orientalis* (L.) L. aqueous bark extract on the average body weight of rats. Table S5: effect of subchronic oral administration of *Nauclea orientalis* (L.) L. aqueous bark extract on haematological parameters of rats. Table S6: effect of subchronic oral administration of *Nauclea orientalis* (L.) L. aqueous bark extract on biochemical parameters of rats. Table S7: effect of subchronic oral administration of *Nauclea orientalis* (L.) L. aqueous bark extract on absolute and relative organ weight of rats. Table S8: screening of *Nauclea orientalis* (L.) L. aqueous bark extract for cardioprotective effect: histological assessment of reversible histological changes. Figure S1: histological investigation of the effect of subchronic oral administration of *Nauclea orientalis* bark extract in Wistar rats (H&E, 10 × 10). (a) Histological investigation in the control group of rats, (b) histological investigation in the rat group treated with *Nauclea orientalis* bark extract. i: Heart tissue, ii: kidney tissue, iii: liver tissue, iv: lung tissue, v: small intestine tissue, and vi: spleen tissue.
