# Supplementary material for: Nauclea orientalis (L.) Bark Extract Protects Rat Cardiomyocytes from Doxorubicin-Induced Oxidative Stress, Inflammation, Apoptosis, and DNA Fragmentation
Source: Oxid Med Cell Longev. 2022 Feb 14;2022:1714841. doi: 10.1155/2022/1714841 (PMC8860544; doi:10.1155/2022/1714841)
Supplement: Supplementary Materials — Table S1: physicochemical properties and phytochemical analysis of Nauclea orientalis bark. Table S2: total polyphenol content and the in vitro antioxidant activity of aqueous bark extract of Nauclea orientalis (L.) L. bark. Table S3: dose-response effect on reversible histological changes of cardiac tissues of Wistar rats exposed to different doses of Nauclea orientalis bark extracts. Table S4: effect of subchronic oral administration of Nauclea orientalis (L.) L. aqueous bark extract on the average body weight of rats. Table S5: effect of subchronic oral administration of Nauclea orientalis (L.) L. aqueous bark extract on haematological parameters of rats. Table S6: effect of subchronic oral administration of Nauclea orientalis (L.) L. aqueous bark extract on biochemical parameters of rats. Table S7: effect of subchronic oral administration of Nauclea orientalis (L.) L. aqueous bark extract on absolute and relative organ weight of rats. Table S8: screening of Nauclea orientalis (L.) L. aqueous bark extract for cardioprotective effect: histological assessment of reversible histological changes. Figure S1: histological investigation of the effect of subchronic oral administration of Nauclea orientalis bark extract in Wistar rats (H&E, 10 × 10). (a) Histological investigation in the control group of rats, (b) histological investigation in the rat group treated with Nauclea orientalis bark extract. i: Heart tissue, ii: kidney tissue, iii: liver tissue, iv: lung tissue, v: small intestine tissue, and vi: spleen tissue. [file 1714841.f1.zip › Supplementary Figure 1.docx]

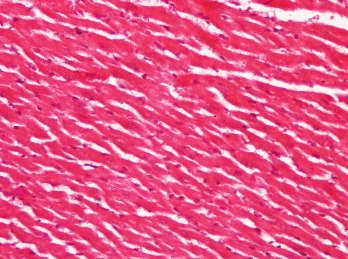

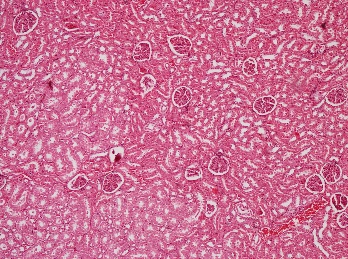

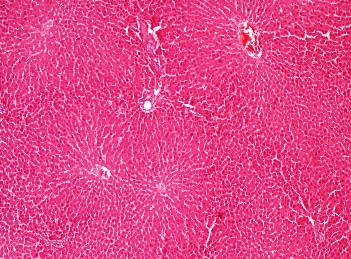

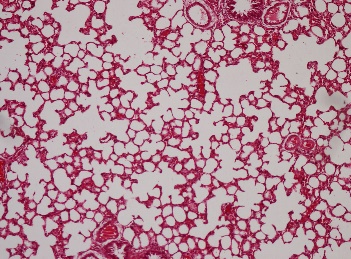


**iii**

**iv**

**ii**

**i**


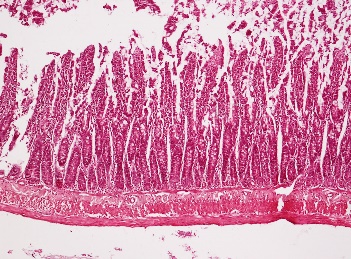

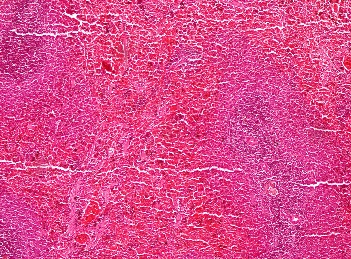


**vi**

**v**

(a)

**iii**


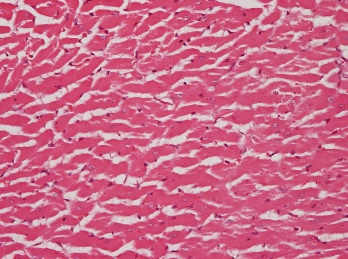

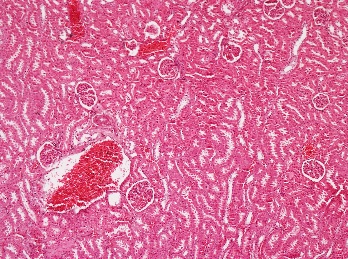

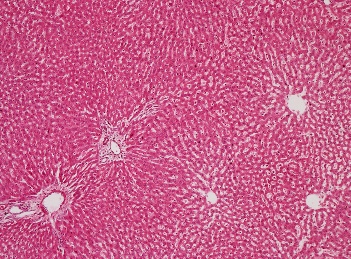

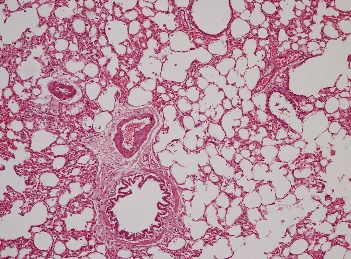


**iv**

**ii**

**i**


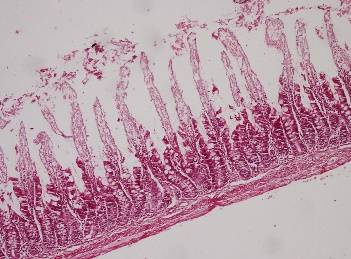

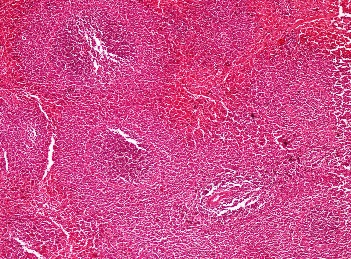


**vi**

**v**

(b)

SUPPLEMENTARY FIGURE 1: Histological investigation of the effect of sub-chronic oral administration of *Nauclea orientalis* bark extract in Wistar rats (H & E, 10×10). (a) Histological investigation in control group of rats, (b) Histological investigation in rat group treated with *Nauclea orientalis* bark extract.

i; Heart tissue, ii; Kidney tissue, iii; Liver tissue, iv; Lung tissue, v; Small intestine tissue, vi; Spleen tissue.
