# Supplementary material for: Nauclea orientalis (L.) Bark Extract Protects Rat Cardiomyocytes from Doxorubicin-Induced Oxidative Stress, Inflammation, Apoptosis, and DNA Fragmentation
Source: Oxid Med Cell Longev. 2022 Feb 14;2022:1714841. doi: 10.1155/2022/1714841 (PMC8860544; doi:10.1155/2022/1714841)
Supplement: Supplementary Materials — Table S1: physicochemical properties and phytochemical analysis of Nauclea orientalis bark. Table S2: total polyphenol content and the in vitro antioxidant activity of aqueous bark extract of Nauclea orientalis (L.) L. bark. Table S3: dose-response effect on reversible histological changes of cardiac tissues of Wistar rats exposed to different doses of Nauclea orientalis bark extracts. Table S4: effect of subchronic oral administration of Nauclea orientalis (L.) L. aqueous bark extract on the average body weight of rats. Table S5: effect of subchronic oral administration of Nauclea orientalis (L.) L. aqueous bark extract on haematological parameters of rats. Table S6: effect of subchronic oral administration of Nauclea orientalis (L.) L. aqueous bark extract on biochemical parameters of rats. Table S7: effect of subchronic oral administration of Nauclea orientalis (L.) L. aqueous bark extract on absolute and relative organ weight of rats. Table S8: screening of Nauclea orientalis (L.) L. aqueous bark extract for cardioprotective effect: histological assessment of reversible histological changes. Figure S1: histological investigation of the effect of subchronic oral administration of Nauclea orientalis bark extract in Wistar rats (H&E, 10 × 10). (a) Histological investigation in the control group of rats, (b) histological investigation in the rat group treated with Nauclea orientalis bark extract. i: Heart tissue, ii: kidney tissue, iii: liver tissue, iv: lung tissue, v: small intestine tissue, and vi: spleen tissue. [file 1714841.f1.zip › Supplementary table 1.docx]

Supplementary table 1: Physicochemical properties and phytochemical analysis of *Nauclea orientalis* bark

| Physicochemical properties | Results | Phytochemicals | Results |
| --- | --- | --- | --- |
| Moisture content (%) | 13.2 | Saponins | Negative |
| Extractable matter in water (%) |  | Polyphenols | Positive |
| Hot | 27.5 | Alkaloids | Positive |
| Cold | 16.5 | Tannins | Positive |
| Extractable matter in methanol (%) |  | Flavonoids | Negative |
| Hot | 9.6 | Anthracene glycosides | Negative |
| Cold | 5.0 | Cyanogenic glycosides | Negative |
| Heavy metals |  | Cardenoloid glycosides | Negative |
| Lead | Not detected | Reducing sugars | Positive |
| Cadmium | Not detected | Proteins | Negative |
| Arsenic | Not detected |  |  |
| Mercury | Not detected |  |  |
|  |  |  |  |
| Microscopic analysis | Parenchyma cells embedded starch grains, thin walled fibres, pitted sclenrenchyma, brown colouring matter, simple and compound starch grains observed |  |  |
