# Supplementary material for: Nauclea orientalis (L.) Bark Extract Protects Rat Cardiomyocytes from Doxorubicin-Induced Oxidative Stress, Inflammation, Apoptosis, and DNA Fragmentation
Source: Oxid Med Cell Longev. 2022 Feb 14;2022:1714841. doi: 10.1155/2022/1714841 (PMC8860544; doi:10.1155/2022/1714841)
Supplement: Supplementary Materials — Table S1: physicochemical properties and phytochemical analysis of Nauclea orientalis bark. Table S2: total polyphenol content and the in vitro antioxidant activity of aqueous bark extract of Nauclea orientalis (L.) L. bark. Table S3: dose-response effect on reversible histological changes of cardiac tissues of Wistar rats exposed to different doses of Nauclea orientalis bark extracts. Table S4: effect of subchronic oral administration of Nauclea orientalis (L.) L. aqueous bark extract on the average body weight of rats. Table S5: effect of subchronic oral administration of Nauclea orientalis (L.) L. aqueous bark extract on haematological parameters of rats. Table S6: effect of subchronic oral administration of Nauclea orientalis (L.) L. aqueous bark extract on biochemical parameters of rats. Table S7: effect of subchronic oral administration of Nauclea orientalis (L.) L. aqueous bark extract on absolute and relative organ weight of rats. Table S8: screening of Nauclea orientalis (L.) L. aqueous bark extract for cardioprotective effect: histological assessment of reversible histological changes. Figure S1: histological investigation of the effect of subchronic oral administration of Nauclea orientalis bark extract in Wistar rats (H&E, 10 × 10). (a) Histological investigation in the control group of rats, (b) histological investigation in the rat group treated with Nauclea orientalis bark extract. i: Heart tissue, ii: kidney tissue, iii: liver tissue, iv: lung tissue, v: small intestine tissue, and vi: spleen tissue. [file 1714841.f1.zip › Supplementary table 2.docx]

Supplementary table 2: Total polyphenol content and the *in vitro* antioxidant activity of aqueous bark extract of *Nauclea orientalis* *(L.)* L. bark

|  | Aqueous bark extract of *Nauclea orientalis* | Standard (Ascorbic acid) |
| --- | --- | --- |
| Total polyphenol content (mg GAE/g dw) | 8.85 ± 2.49 | NA |
| IC_50_ value in DPPH assay (µg/mL) | 135.79 ± 0.13 | 7.65 ± 0.32 |
| IC_50_ value in NO inhibition assay (µg/mL) | 197.47 ± 0.07 | 33.42 ± 0.18 |
| FRAP value (µM) | 10.33 ± 1.30 | NA |
| DPPH; 2,2-diphenyl-1-picrylhydrazyl, No; Nitric oxide, FRAP; Ferric reducing antioxidant power, NA; Not applicable  IC_50_ (concentration of the plant extract required to inhibit DPPH/NO radical formation by 50%) obtained by linear regression analysis | | |
