# Supplementary material for: Nauclea orientalis (L.) Bark Extract Protects Rat Cardiomyocytes from Doxorubicin-Induced Oxidative Stress, Inflammation, Apoptosis, and DNA Fragmentation
Source: Oxid Med Cell Longev. 2022 Feb 14;2022:1714841. doi: 10.1155/2022/1714841 (PMC8860544; doi:10.1155/2022/1714841)
Supplement: Supplementary Materials — Table S1: physicochemical properties and phytochemical analysis of Nauclea orientalis bark. Table S2: total polyphenol content and the in vitro antioxidant activity of aqueous bark extract of Nauclea orientalis (L.) L. bark. Table S3: dose-response effect on reversible histological changes of cardiac tissues of Wistar rats exposed to different doses of Nauclea orientalis bark extracts. Table S4: effect of subchronic oral administration of Nauclea orientalis (L.) L. aqueous bark extract on the average body weight of rats. Table S5: effect of subchronic oral administration of Nauclea orientalis (L.) L. aqueous bark extract on haematological parameters of rats. Table S6: effect of subchronic oral administration of Nauclea orientalis (L.) L. aqueous bark extract on biochemical parameters of rats. Table S7: effect of subchronic oral administration of Nauclea orientalis (L.) L. aqueous bark extract on absolute and relative organ weight of rats. Table S8: screening of Nauclea orientalis (L.) L. aqueous bark extract for cardioprotective effect: histological assessment of reversible histological changes. Figure S1: histological investigation of the effect of subchronic oral administration of Nauclea orientalis bark extract in Wistar rats (H&E, 10 × 10). (a) Histological investigation in the control group of rats, (b) histological investigation in the rat group treated with Nauclea orientalis bark extract. i: Heart tissue, ii: kidney tissue, iii: liver tissue, iv: lung tissue, v: small intestine tissue, and vi: spleen tissue. [file 1714841.f1.zip › Supplementary table 5.docx]

Supplementary table 5: Effect of sub-chronic oral administration of *Nauclea orientalis* (L.) L. aqueous bark extract on haematological parameters of rats

| Haematological parameters | Control group | Rats treated with *N. orientalis* bark extract (2 g/kg) |
| --- | --- | --- |
| WBC (×10³/µL) | 3.8± 0.3 | 4.2±0.4 |
| RBC (×10⁶/µL) | 8.1±0.1 | 8.2±0.1 |
| Haemoglobin (g/dL) | 15.0±0.3 | 15.4±0.2 |
| Haematocrit (%) | 52.7±1.2 | 53.9±1.0 |
| MCV (fL) | 65.2±0.9 | 65.8±0.5 |
| MCH (pg) | 18.6±0.1 | 18.8±0.1 |
| MCHC (g/L) | 28.6±0.4 | 28.7±0.4 |
| Platelets (×10³/µL) | 695.0±22.0 | 689.2±34.0 |
| WBC; white blood cells, RBC; red blood cells, MCV; mean corpuscular cell volume, MCH; mean corpuscular haemoglobin, MCHC; mean corpuscular haemoglobin concentration. All values are expressed as mean ± SEM (n=10). | | |
