# Supplementary material for: Nauclea orientalis (L.) Bark Extract Protects Rat Cardiomyocytes from Doxorubicin-Induced Oxidative Stress, Inflammation, Apoptosis, and DNA Fragmentation
Source: Oxid Med Cell Longev. 2022 Feb 14;2022:1714841. doi: 10.1155/2022/1714841 (PMC8860544; doi:10.1155/2022/1714841)
Supplement: Supplementary Materials — Table S1: physicochemical properties and phytochemical analysis of Nauclea orientalis bark. Table S2: total polyphenol content and the in vitro antioxidant activity of aqueous bark extract of Nauclea orientalis (L.) L. bark. Table S3: dose-response effect on reversible histological changes of cardiac tissues of Wistar rats exposed to different doses of Nauclea orientalis bark extracts. Table S4: effect of subchronic oral administration of Nauclea orientalis (L.) L. aqueous bark extract on the average body weight of rats. Table S5: effect of subchronic oral administration of Nauclea orientalis (L.) L. aqueous bark extract on haematological parameters of rats. Table S6: effect of subchronic oral administration of Nauclea orientalis (L.) L. aqueous bark extract on biochemical parameters of rats. Table S7: effect of subchronic oral administration of Nauclea orientalis (L.) L. aqueous bark extract on absolute and relative organ weight of rats. Table S8: screening of Nauclea orientalis (L.) L. aqueous bark extract for cardioprotective effect: histological assessment of reversible histological changes. Figure S1: histological investigation of the effect of subchronic oral administration of Nauclea orientalis bark extract in Wistar rats (H&E, 10 × 10). (a) Histological investigation in the control group of rats, (b) histological investigation in the rat group treated with Nauclea orientalis bark extract. i: Heart tissue, ii: kidney tissue, iii: liver tissue, iv: lung tissue, v: small intestine tissue, and vi: spleen tissue. [file 1714841.f1.zip › Supplementary table 6.docx]

Supplementary table 6: Effect of sub-chronic oral administration of *Nauclea orientalis* (L.) L. aqueous bark extract on biochemical parameters of rats

| Biochemical parameters | Control group | Rats treated with *N. orientalis* bark extract (2 g/kg) |
| --- | --- | --- |
| Liver function tests |  |  |
| AST (U/L) | 30.1±0.7 | 32.7±2.8 |
| ALT (U/L) | 51.7±2.3 | 50.1±1.6 |
| ALP (U/L) | 117.0±6.3 | 123.2±5.5 |
| Kidney function tests |  |  |
| Creatinine (µmol/L) | 68.9±1.6 | 70.8±4.2 |
| Urea (mg/dL) | 42.4±1.0 | 39.0±1.6 |
| AST; aspartate amino transferase, ALT; alanine amino transferase, ALP; alkaline phosphatase. All values are expressed as mean ± SEM (n=10). | | |
