# Supplementary material for: Nauclea orientalis (L.) Bark Extract Protects Rat Cardiomyocytes from Doxorubicin-Induced Oxidative Stress, Inflammation, Apoptosis, and DNA Fragmentation
Source: Oxid Med Cell Longev. 2022 Feb 14;2022:1714841. doi: 10.1155/2022/1714841 (PMC8860544; doi:10.1155/2022/1714841)
Supplement: Supplementary Materials — Table S1: physicochemical properties and phytochemical analysis of Nauclea orientalis bark. Table S2: total polyphenol content and the in vitro antioxidant activity of aqueous bark extract of Nauclea orientalis (L.) L. bark. Table S3: dose-response effect on reversible histological changes of cardiac tissues of Wistar rats exposed to different doses of Nauclea orientalis bark extracts. Table S4: effect of subchronic oral administration of Nauclea orientalis (L.) L. aqueous bark extract on the average body weight of rats. Table S5: effect of subchronic oral administration of Nauclea orientalis (L.) L. aqueous bark extract on haematological parameters of rats. Table S6: effect of subchronic oral administration of Nauclea orientalis (L.) L. aqueous bark extract on biochemical parameters of rats. Table S7: effect of subchronic oral administration of Nauclea orientalis (L.) L. aqueous bark extract on absolute and relative organ weight of rats. Table S8: screening of Nauclea orientalis (L.) L. aqueous bark extract for cardioprotective effect: histological assessment of reversible histological changes. Figure S1: histological investigation of the effect of subchronic oral administration of Nauclea orientalis bark extract in Wistar rats (H&E, 10 × 10). (a) Histological investigation in the control group of rats, (b) histological investigation in the rat group treated with Nauclea orientalis bark extract. i: Heart tissue, ii: kidney tissue, iii: liver tissue, iv: lung tissue, v: small intestine tissue, and vi: spleen tissue. [file 1714841.f1.zip › Supplementary table 7.docx]

Supplementary table 7: Effect of sub-chronic oral administration of *Nauclea orientalis* (L.) L. aqueous bark extract on absolute and relative organ weight of rats

| Organ | Absolute organ weight (g) | | Relative organ weight (g per 100 g body weight) | |
| --- | --- | --- | --- | --- |
|  | Control group | Rats treated with *Nauclea orientalis* bark extract (2 g/kg) | Control group | Rats treated with *Nauclea orientalis* bark extract (2 g/kg) |
| Heart | 0.8±0.0 | 0.7±0.0 | 0.3±0.0 | 0.3±0.0 |
| Kidneys | 1.4±0.0 | 1.4±0.0 | 0.5±0.0 | 0.5±0.0 |
| Liver | 7.4±0.1 | 7.5±0.1 | 2.6±0.0 | 2.7±0.0 |
| Lungs | 1.1±0.1 | 1.1±0.0 | 0.4±0.0 | 0.4±0.0 |
| Small intestine | 5.7±0.3 | 5.9±0.4 | 2.0±0.1 | 2.2±0.2 |
| Spleen | 0.5±0.0 | 0.5±0.0 | 0.2±0.0 | 0.2±0.0 |
| All values are expressed as mean ± SEM (n=10). | | | | |
